# Impact of illumination spectrum and eye pigmentation on image quality from a fundus camera using transscleral illumination

**DOI:** 10.1117/1.JBO.26.7.076003

**Published:** 2021-07-08

**Authors:** Alexey Stepanov, Jostein Thorstensen, Jon Tschudi

**Affiliations:** SINTEF Digital, Oslo, Norway

**Keywords:** fundus camera, eye pigmentation, transscleral illumination, multispectral retinal imaging

## Abstract

**Significance:** The use of the transscleral illumination approach has the potential to simplify the optical design of fundus cameras. In particular, this approach could allow the use of smaller and cheaper cameras that are easier to use by non-specialists, thereby facilitating a wider spread of eye disease screening programs.

**Aim:** Our aim was to investigate the suitability of transscleral illumination in a fundus camera system. In particular, we explored the impact of the illumination spectrum and the eye pigmentation on the quality of the image. These factors have never been systematically investigated before in the literature on transscleral illumination.

**Approach:** A fundus camera was constructed using transscleral illumination. We studied the influence of eye pigmentation and choice of illumination spectra on the image quality for a group of 10 individuals with varied skin pigmentation, ranging from pale white (North-European) to darkest brown (African). The influence of the light source spectrum on the image quality was assessed using wavelength filters.

**Results:** There was a difference of a factor of 100 in the signal level of retinal images between individuals with low and high skin pigmentation. The image contrast was highest using illumination wavelengths of 500 to 600 nm. The illumination level can be adjusted to obtain high-quality images for highly pigmented eyes while keeping the system eye-safe.

**Conclusions:** We have demonstrated that a fundus camera with transscleral illumination can provide high-quality images. However, the variations observed in scleral and retinal pigmentation in a practical setting require a system that must be able to adapt illumination and/or exposure to the individual patient.

## 1 Introduction

A number of common eye diseases can be diagnosed using retina images. A prominent example is diabetic retinopathy, which is a leading cause of vision loss in many developed countries.^1^ Other examples include glaucoma,^2^ age-related macular degeneration,^3^ retinopathy of prematurity,^4^ and malarial retinopathy.^5^ Early screening of these diseases can simplify treatment and prevent complications leading to blindness.^6^ Currently, this type of screening is performed at hospitals or other specialized institutions using expensive fundus camera systems that capture high-resolution retina images. In countries where this type of healthcare service is not available for the majority of the population, such screening is simply not possible. Therefore, a compact, handheld, low-cost, and easy-to-use device that enables early screening, would increase the accessibility of this technology and enable widespread early diagnosis.

The most common method to construct a fundus camera is to both illuminate and image through the pupil. This principle is widely used in stationary fundus cameras and provides high-quality images, but it has several drawbacks: complicated optics are needed to avoid reflections (the same lens is used both for imaging and illumination), a small imaging aperture provides a limited field-of-view, mydriatic dilation of the pupil is often required before imaging, and it is bulky in size.^7^ Several new approaches have been proposed to address these issues.^8^

One possible way to simplify the optical design and make such cameras more compact is to separate the illumination and imaging optical paths such that the retina is not illuminated through the pupil. Various illumination paths have been investigated, such as transscleral (both contact and contactless),^4^^,^^9^^–^^12^ transpalpebral,^13^ and transcranial.^14^ These approaches obtain a wider field-of-view, eliminate superficial specular reflections, illuminate the fundus uniformly due to a highly scattering illumination path, and allow for simpler optical design and larger tolerances in optical components.^9^ The latter points are especially important for a compact, low-cost, handheld camera for early screening. However, because these methods illuminate through highly scattering and absorptive media, they require more power for the illumination of the retina.

Recent research on transscleral illumination designs^10^^,^^11^ has shown that this approach enables the design of compact devices with sufficient image quality. However, these systems have not been tested on large groups of people, and some physiological parameters have significant impact on the applicability of this approach. The most critical parameter is the total eye pigmentation, which is the combined pigmentation in the choroid and retinal pigment epithelium. This parameter differs widely across the population^15^ and affects the transmission of light through the sclera, thereby significantly influencing the optical signal levels and the resulting quality of the images. Toslak et al.^16^ obtained retinal images using trans-pars-planar illumination from a subject with high pigmentation, but they do not provide further details on pigmentation level or quantitative influence of pigmentation on signal levels. The choice of the illumination source (especially its wavelength) also has an effect on image quality and contrast, which is important for detection of specific diseases (e.g., diabetic retinopathy^17^).

To evaluate the impact of eye pigmentation and the illumination spectrum on retina image quality, a fundus camera with transscleral illumination was constructed, and experiments were performed using different illumination spectra. Individuals with different eye pigmentation were examined; however, eye pigmentation levels were not independently evaluated. The results show that the illumination spectrum strongly influences the image contrast, and that differences in eye pigmentation cause differences in signal levels; in particular, the signal level between eyes with the lowest and highest pigmentation differs by a factor of 100. Both of these findings are crucial for the design of a fundus camera with transscleral illumination.

## 2 Method

### 2.1 Optical Setup

**Fig. 1:**
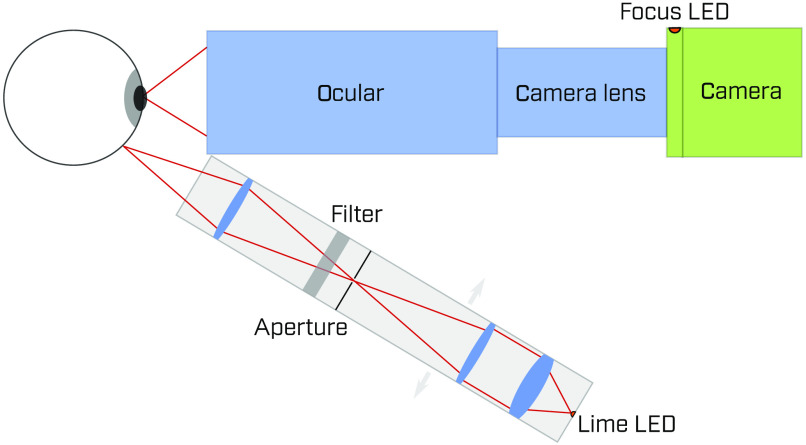
Optical setup with adjustable illumination.

**Fig. 2:**
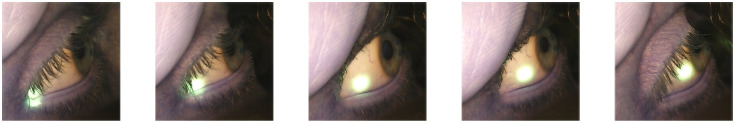
Different light source positions. These images are captured synchronously with retinal images, to document the illumination position. It is worthy to note that due to over-exposure of the illumination, the spot appears larger than its actual size.

The optical setup used in this work is shown in Fig. 1. The retina is imaged by a camera with a camera lens in combination with an ocular. We used non-contact transscleral illumination, wherein the LED illumination is imaged onto the sclera at an angle of ∼30  deg relative to the optical axis of the camera system. The position of the LED spot on the sclera can be adjusted, as shown in Fig. 2, to investigate the image quality as a function of the spot position.

The positioning of the eye relative to the entrance pupil of the ocular, as well as the correct focusing of the system, are critical for good imaging. To ensure the correctness of these parameters, an LED in a spacer ring was placed in front of the camera sensor to illuminate the sensor chip such that a faint pixel grid is observable to the subject when the eye is in the correct position and the optical system is in focus. The subject uses a chin rest to maintain the steady position of their head.

**Fig. 3:**
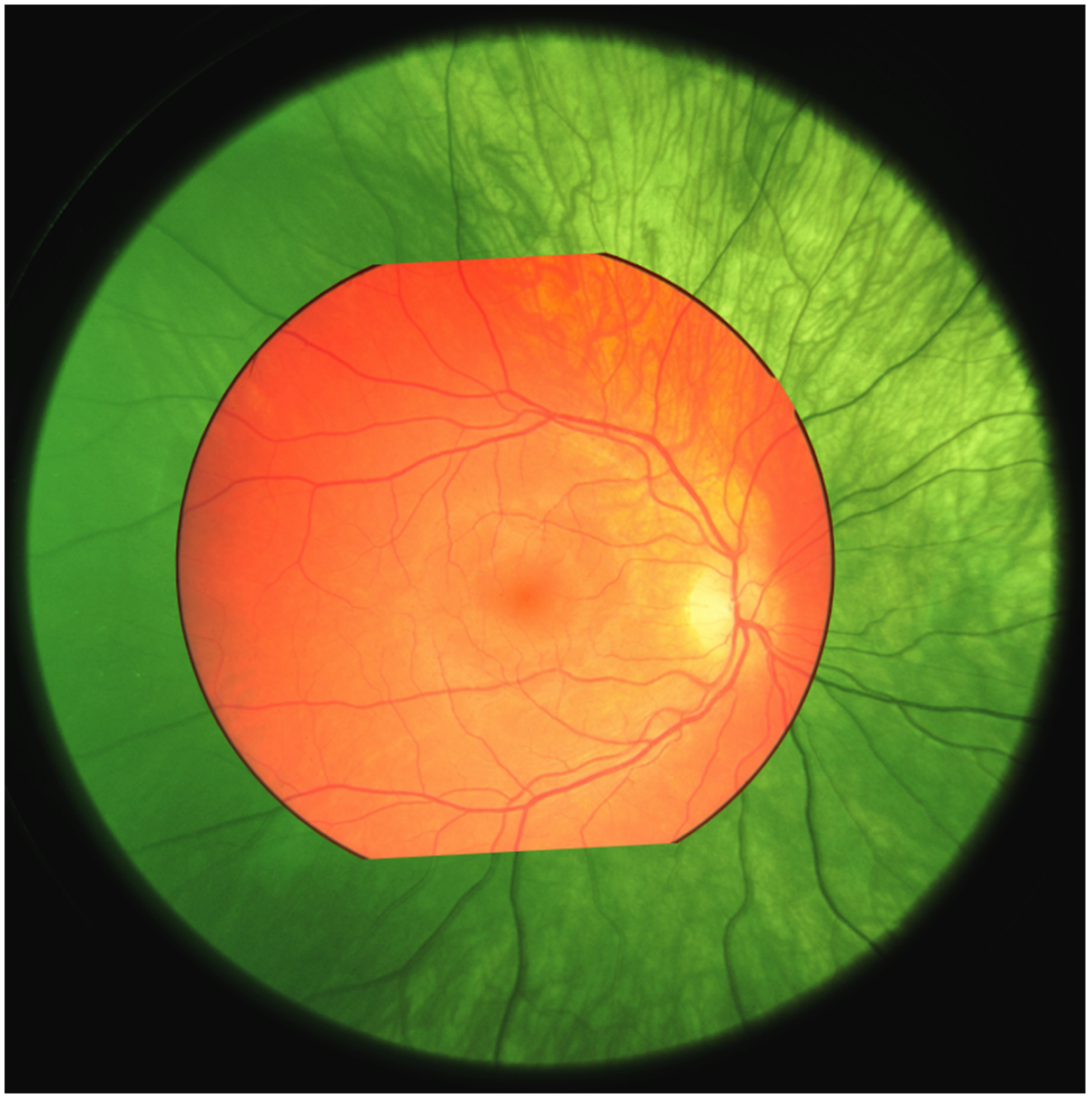
Fundus image taken with Zeiss Visucam PRO NM (red) superimposed on image taken with our setup (green).

**Fig. 4:**
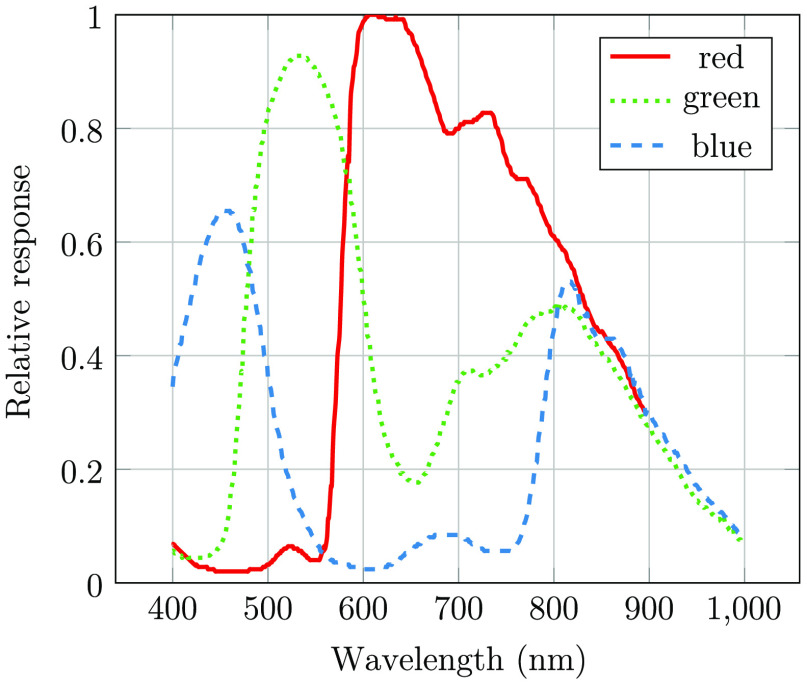
Camera spectral response in different channels.

We used a DBK 33UX264.AS USB 3.0 color camera from The Imaging Source (2448×2048  pixels), which was fitted with a Kowa 50 mm f/2.8 lens and a Kowa TE-11WZ ocular with 17-mm eye relief. The illumination has a working distance of 40 mm. We used a Chiyi CS1002-PF camera to capture images of the sclera and the illumination spot. The capture was synchronized with retina image capture to document illumination position for all retina images. In Fig. 3, we compare the field-of-view of our retina camera with a fundus image taken with a professional camera Zeiss Visucam PRO NM, with 45-deg external field angle. From this comparison, we estimated the field-of-view of our camera to be 70-deg external angle (or 105 degrees eye angle). With 1400 pixels within the field-of-view, we estimated our resolution to be ∼15  μm (based on an eye radius of 11 mm). The relative spectral response of the camera sensor is shown in Fig. 4.

**Fig. 5:**
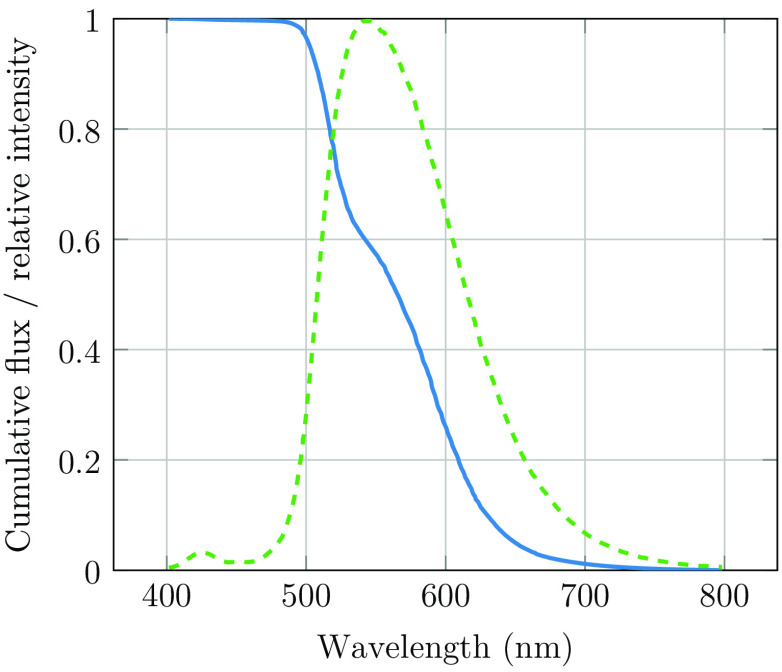
Cumulative relative photon flux (blue, solid line) and spectrum (green, dashed line) from the lime LED.

For the illumination, we used a $1\times 1\;{\mathrm{mm}}^{2}$ LUXEON Rebel Lime LED (with a spectrum centered around 560 nm). The illumination path is a 3:1 imaging of the LED onto the sclera. The normalized LED spectral photon flux is shown in Fig. 5 along with the photon flux integrated from long wavelengths toward shorter wavelengths. Therefore, the y-value represents the photon flux that passes through a longpass filter.

Bartczak et al.^17^ investigated the influence of the illumination spectra on the contrast and quality of fundus images. They optimized the illumination spectrum to increase the contrast of a variety of features on the retina. It is worth noting that most features require illumination at wavelengths between 500 and 600 nm for optimum contrast. Additionally, Toslak et al.^16^ investigated the differences in observable features in retina images illuminated with LEDs of different central wavelength.

Considering transpupilar illumination (utilized by traditional fundus cameras), the illumination spectrum (source spectrum) is the same as the spectrum incident on the retina. However, in the case of transscleral illumination (our system), the illumination will experience significant absorption, particularly through the choroid and retinal pigment epithelium layers. This absorption will be strongest in the short-wavelength region. Therefore, a green or lime LED may be a good starting point for transscleral illumination. Toslak et al.^13^ used a lime LED in their study and reported a slightly red-dominated transmission spectrum.

A set of long-, short-, and band-pass filters were used in the illumination path to systematically investigate the image contrast obtained from various wavelength bands.

### 2.2 Eye Safety

For eye safety calculations, we used ISO 15004-2:2007—Ophthalmic instruments—Part 2: Light hazard protection. This standard provides separate limits for the anterior parts of the eye and for the retina, for photochemical damage (short wavelengths) and thermal damage (primarily longer wavelength) for different wavelengths, pulse durations, etc. To calculate the exposure levels on the anterior parts of the eye, we measured the power incident on the sclera to be 80 mW on a 2.8×2.8  mm rectangle. Our illumination is only switched on during the exposure, with a pulse duration of 150 ms. For exposure levels on the retina, we assume, as an absolute worst-case scenario, that the light passes un-attenuated through the sclera, maintaining the illuminated area when reaching the retina.

According to the ISO standard, a maximum of $10\;{J/\mathrm{cm}}^{2}$ weighted irradiance is allowed on the retina to avoid concerns related to photochemical hazard. To evaluate the retinal photochemical hazard, one needs to integrate over the illumination power spectrum weighted by the aphakic photochemical hazard weighting function A

$$H_{A-R}=\sum E_{\lambda }\cdot t\cdot A(\lambda )\cdot \Delta \lambda .$$

For the calculation of the retinal photochemical hazard, the LED spectrum shown in Fig. 5 is weighted with the photochemical hazard weighting function from the ISO standard. Since the lime LED is a phosphor-converted blue LED, we observe a slight blue remnant at approximately 420 nm. At these wavelengths, the hazard weight is ∼100 times greater than that at 550 nm, and therefore, the low power at this short-wavelength actually dominates the photochemical hazard. Thus, it is not sufficient to base the hazard calculations on the peak wavelength of the LED. To minimize the photochemical hazard while maximizing light throughput, a 500-nm longpass filter was placed in front of the LED. By integrating the product of the filtered LED power spectrum and the hazard function, we determined an equivalent exposure level of $1.9\;{\mathrm{mJ}/\mathrm{cm}}^{2}$. Comparing with the exposure limit of $10\;{J/\mathrm{cm}}^{2}$, the eye can thus be exposed more than 5000 times without reaching the limit exposure.

Retinal thermal hazard is calculated similarly. The limit for pulsed instruments is given by $\frac{10}{d_{r}}\cdot t^{3/4}\;{J/\mathrm{cm}}^{2}=1.4\;{J/\mathrm{cm}}^{2}$. The retinal thermal exposure is calculated by weighting the illumination power spectrum with the thermal hazard weighting function R, which for our wavelengths is equal to 1:

$$H_{\mathrm{VIR}-R}=\sum E_{\lambda }\cdot \Delta \cdot t\cdot R(\lambda )\cdot \Delta \lambda .$$

In our case, $H_{\mathrm{VIR}-R}=0.16\;{J/\mathrm{cm}}^{2}$. Finally, the anterior segment visible and infrared radiation radiant exposure limit is $25\cdot t^{0.25}\;{J/\mathrm{cm}}^{2}=15.5\;{J/\mathrm{cm}}^{2}$, and the exposure is given as

$$H_{\mathrm{VIR}-\mathrm{AS}}=\sum H_{\lambda }\cdot \Delta \lambda .$$

In our case, $H_{\mathrm{VIR}-\mathrm{AS}}=0.16\;{J/\mathrm{cm}}^{2}$. The applicable limit values and the measured/simulated exposure levels are listed in Table 1. In our experiments with bandpass-filtered illumination, we use the same LED, but selectively removed parts of the illumination spectrum by filtering. In this process, optical power, and thereby exposure level get reduced. Thus, our bandpass-filtered illumination is eye-safe.

**Table 1:** Exposure levels and safety limits according to ISO 15004-2:2007.

	Limit ($J/{\mathrm{cm}}^{2}$)	Exposure level
Anterior thermal	15.5	$0.16\;{J/\mathrm{cm}}^{2}$
Retinal thermal	1.4	$0.16\;{J/\mathrm{cm}}^{2}$
Retinal photochemical	10	$1.9\;{\mathrm{mJ}/\mathrm{cm}}^{2}$

From the table, we observe that the measured and estimated exposure values are far lower than the specified limits, even with our worst-case scenario of zero attenuation and beam expansion for the retinal limits. Therefore, it was concluded that the system is eye-safe.

### 2.3 Study Subjects and Procedure

We recruited 10 healthy adults with different skin pigmentations under the assumption that eye and skin pigmentation are correlated;^15^ see Table 2. The eye pigmentation is the combined pigmentation in the choroid and retinal pigment epithelium. The study was pre-approved by the Norwegian Centre for Research Data and conducted in accordance with the Declaration of Helsinki with written consent acquired from each subject before the study. Beyond age, sex, and skin pigmentation, no personal or any other data was collected. The skin pigmentation was qualified using the Fitzpatrick scale.^18^ The range 1 to 6 covers skin types from pale white (North-European) to the darkest brown (African).

**Table 2:** Test subject statistics.

Age, mean (standard deviation)	38.8 (5.23)
Number of subjects (male/female)	10 (6/4)
Eye color (number of subjects)	dark brown (3), brown (6), blue (1)
Skin pigmentation (number of subjects)	6 (1), 5 (2), 4 (1), 3 (2), 2 (3), 1 (1)

**Fig. 6:**
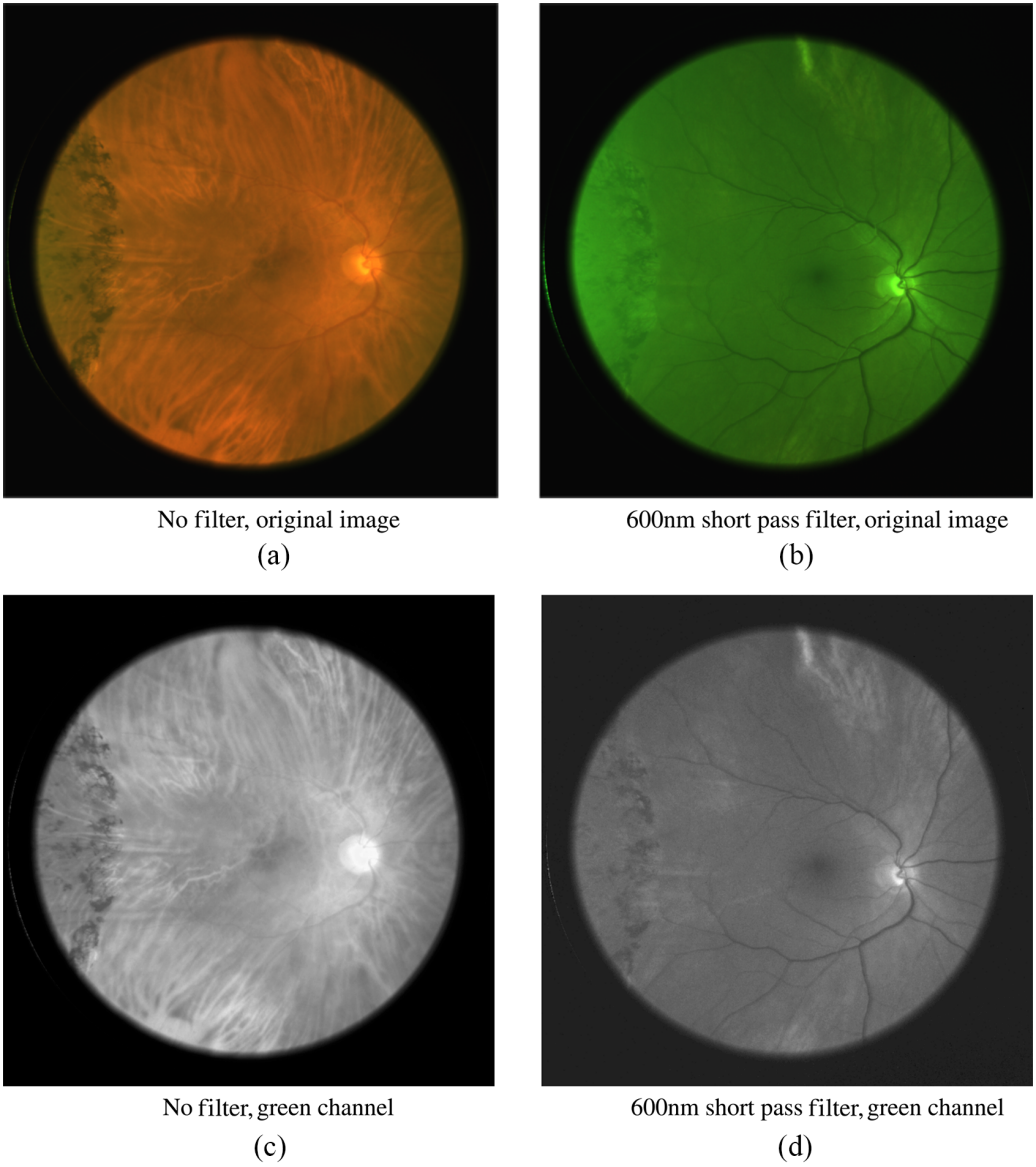
Images taken without a filter [panels (a) and (c)] and with a 600-nm shortpass filter [panels (b) and (d)]. The blood vessels show a higher contrast in the shortpass filtered image. Note that when comparing the green camera channel (bottom row), (c) the un-filtered and (d) filtered images show very different information content.

For each subject, several images were captured using different wavelength filters in the illumination path. Sample images of the retina are shown in Fig. 6. We varied the position of the illumination spot on the sclera from the edge of the cornea to the corner of the eye (see Fig. 2). The analog gain of the sensor was varied to obtain usable signal levels from eyes with a high pigmentation level.

For each image, we computed the average signal level from the optical disk and the contrast ratio between the blood vessels and background. The contrast ratio provides an indication of usable information contained in the images to detect abnormalities. The images were normalized with respect to the analog gain.

Due to a lack of images with disease markers, we used the contrast between blood vessels (arteries and veins) and the background of the retina as a proxy for image quality.

**Fig. 7:**
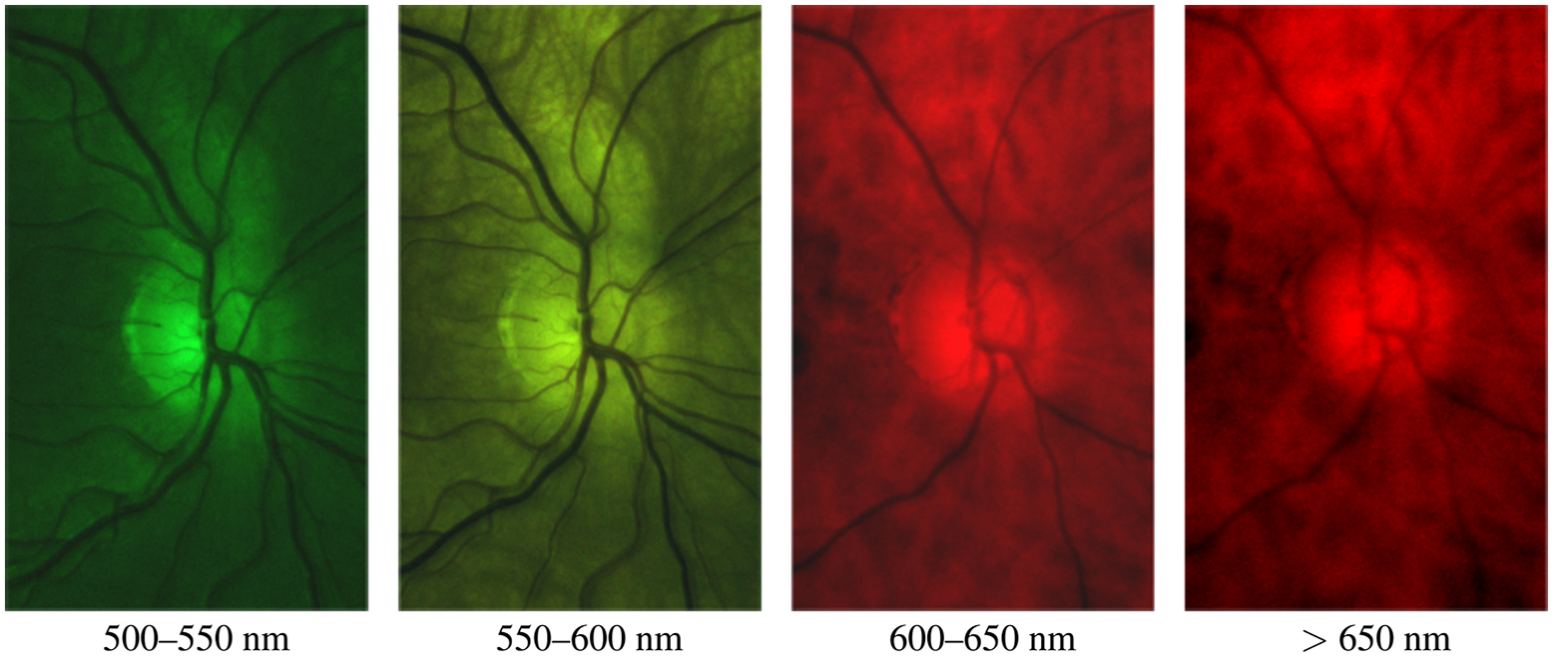
Retina images obtained using different bandpass filters. The images are normalized for presentation.

We obtained several images with different filters (see Fig. 7) and analyzed the image contrast using the following formula:

$$\text{contrast}=\frac{R-V}{\sigma_{V}},$$

where R is the average signal level from the retina (background), V is the average signal from blood vessels, and $\sigma_{V}$ is the standard deviation of signals from the blood vessels. There are many methods to segment blood vessels in retina images.^19^ In these examples, the images were segmented manually as the number of images was low and there was considerable variation in the signal and contrast levels.

## 3 Results and Discussion

### 3.1 Impact of LED Spectra on Image Quality

**Fig. 8:**
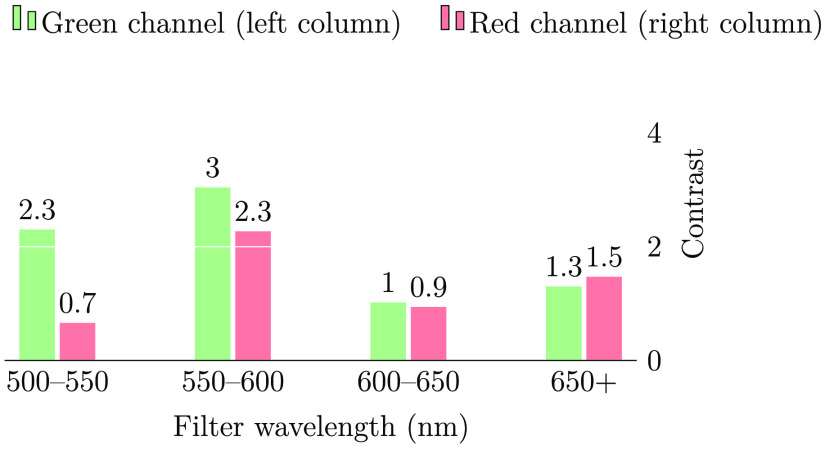
Contrast between the blood vessels and background using different filters. The contrast is high when using wavelengths shorter than 600 nm, wherein the blood attenuation is high (see Fig. 9). The contrast is highest using the green channel, and the response is highest at these wavelengths.

The computed contrast values for the red and green channels of the images are shown in Fig. 8.

**Fig. 9:**
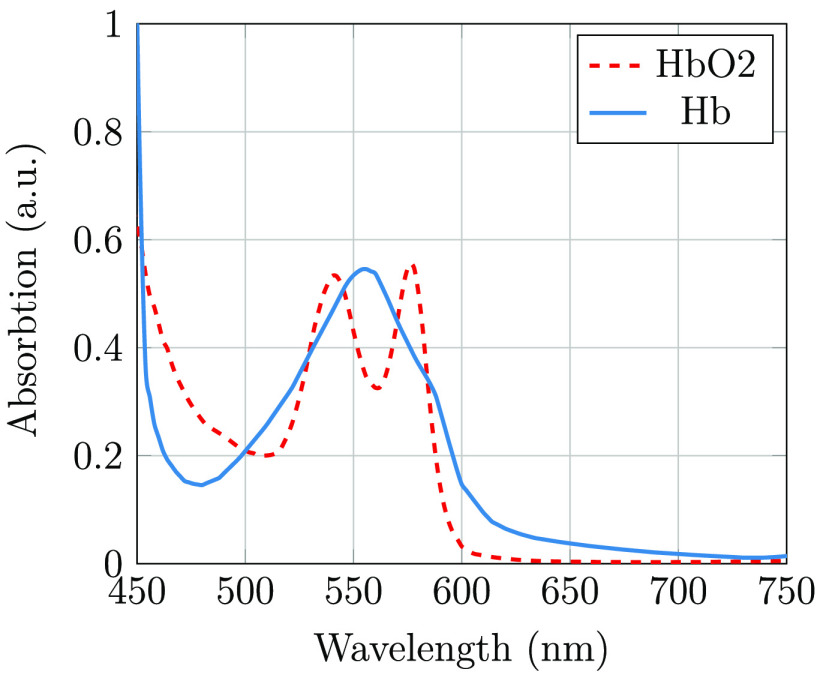
Absorption spectra of oxygenated (${\mathrm{HbO}}_{2}$) and de-oxygenated (Hb) hemoglobin.

We observe that the images illuminated with wavelengths from 500 to 600 nm showed the highest contrast. This agrees with the results reported by Bartczak et al.,^17^ and it is as expected from examining the absorption spectrum of both oxygenated and de-oxygenated hemoglobin (Fig. 9). The contrast measure is highest in the green channel, as this channel has the highest spectral response when illuminating with wavelengths shorter than 600 nm. In Figs. 6(c) and 6(d), we can clearly observe the importance of wavelength filtering. While both of these images are taken using an LED with a peak wavelength of 565 nm (green), and both images show the green channel of the camera, the contents of the two images are radically different. It is quite clear that image (c) is dominated by long-wavelength illumination and thus shows far lower contrast for the blood vessels clearly observable in image (d).

**Fig. 10:**
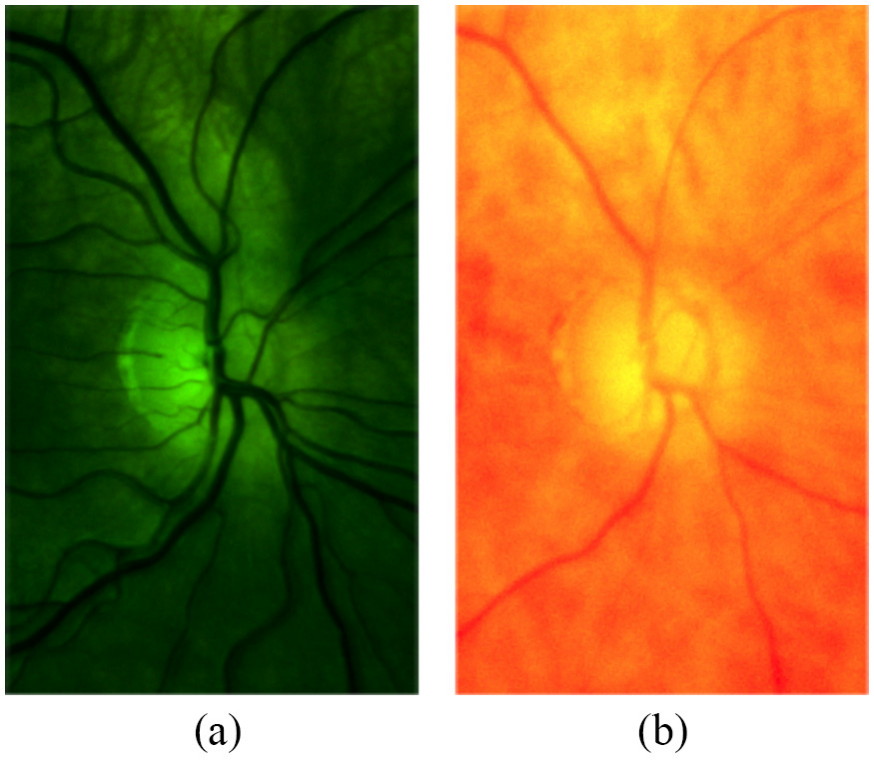
Images obtained with (a) 600-nm shortpass and (b) 600-nm longpass filters, thereby demonstrating the good contrast obtained using wavelengths in which hemoglobin absorption is high.

It is interesting to compare the images captured with the 600-nm short-pass and 600-nm longpass filters (Fig. 10). The shortpass-filtered image shows a high contrast of both the arteries and veins, whereas the longpass-filtered image shows far less contrast and presumably shows only veins as only de-oxygenated hemoglobin absorbs at wavelengths longer than 600 nm.

**Fig. 11:**
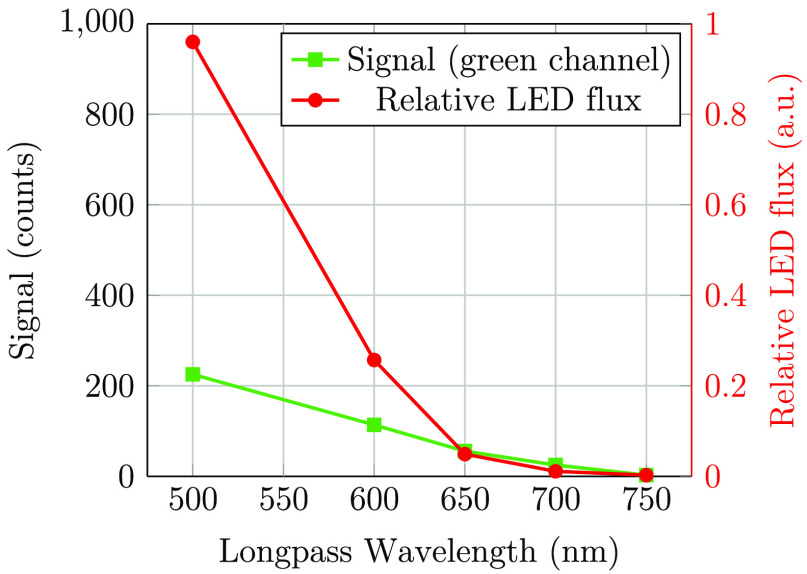
Relative photon flux from the LED (red, circle marks) and the signal in the green channel (green, square marks). We observed that the short-wavelength light is more strongly attenuated than the long-wavelength light.

Figure 11 shows signal levels in a series of images captured with a longpass filter. We plot the signal in the green channel of the camera along with relative photon flux obtained from the LED (Fig. 5) versus the longpass cut-off wavelength. It can be observed that the strong increase in the LED flux at wavelengths shorter than 650 nm leads to only a moderate increase in the received signal as a result of the strong attenuation at shorter wavelengths.

The observed data show that the short-wavelength light is far more strongly attenuated than the long-wavelength light. Even though the green portion of the LED signal is far stronger than the red and infrared, there is only a modest increase in the signal, such as when transitioning from a 600- to a 500-nm longpass filter.

To quantify the influence of wavelength on the LED spectrum after transmission through the sclera, we assume that the camera signal can be described as follows:

S(λ)∝P(λ)·T(λ)·QE(λ),

where S(λ) is the signal level at wavelength λ, P(λ) is the photon flux, T(λ) is the transmission through sclera, and QE(λ) is the camera response. Please note that we did not attempt to extract optical properties such as absorption coefficients or optical path length. The transmission will be affected by both these parameters, with optical path length being dependent on wavelength-dependent scattering. Nevertheless, we can extract valuable information about photon transport through the sclera using the equation above. The relative transmission can be estimated using the below expression:

$$T(\lambda )\propto \frac{S(\lambda )}{P(\lambda )\cdot \mathrm{QE}(\lambda )}$$

**Fig. 12:**
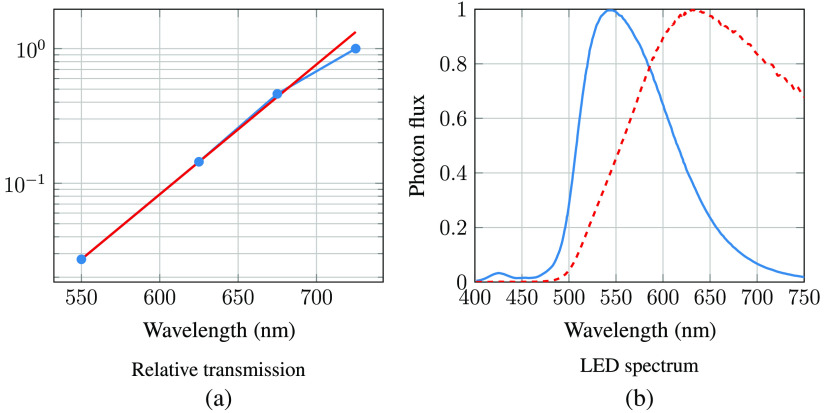
(a) Blue curve with circle marks: Relative transmission through the sclera (normalized to the value at 725 nm) estimated from the LED photon flux and the received signal; see Fig. 11. Red curve: fit to the transmission data: $\alpha e^{\lambda /50}$, where λ is the wavelength of the light source (in nm) and α is a scaling constant. (b) Original LED spectrum (blue, solid line) and modified spectrum after transmission through sclera (red, dashed line).

By taking the derivative of the signals in Fig. 11 and extracting the average quantum efficiency in our wavelength bands from Fig. 4, we may estimate the relative wavelength dependence of transscleral light transmission (Fig. 12). There is approximately a 10-times greater transmission at 650 nm compared with that at 550 nm. An exponential function was fitted to the relative transmission, giving $\alpha e^{\lambda /50}$, where λ is the wavelength of the light source (in nm) and α is a scaling constant. This exponential fit was used to estimate the modified LED spectrum after transmission through the sclera (Fig. 12). While this method provides a rough estimate only, it may be observed that the lime LED has a maximum photon flux in the deep-red region (around 650 nm) after transmission through the sclera, and a significant infrared tail is present. As seen in Figs. 6, 7, and 8, and as discussed by Bartczak et al.,^17^ long-wavelength light will reduce contrast for many disease markers.

### 3.2 Illumination Positioning on Sclera

**Fig. 13:**
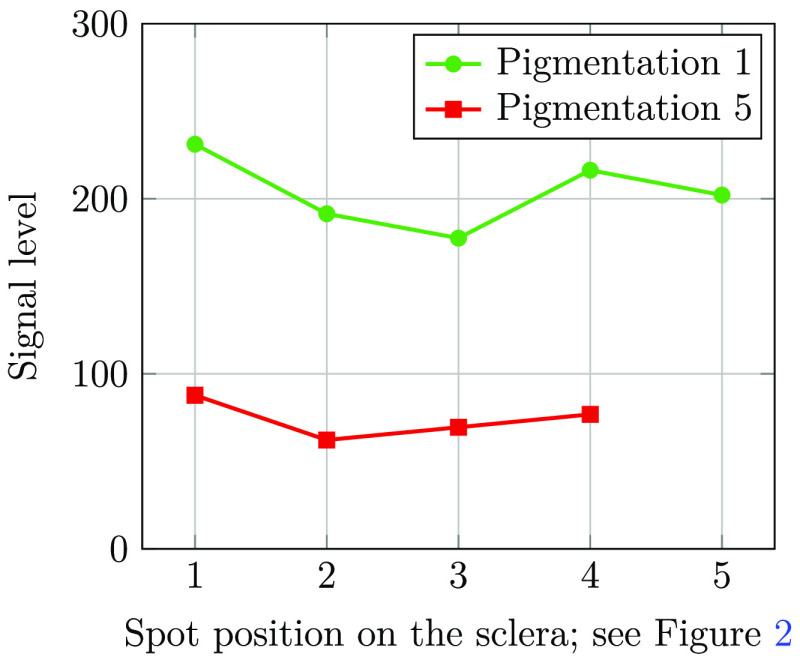
Signal levels as function of position of illumination spot on the sclera. The reported signal levels are after application of digital gain.

Wang et al.^10^ and Toslak et al.^11^ reported fairly significant variations in the transmitted signal as a function of the position of the illuminated spot on the sclera. The width of the pars plana transmission peak reported by Wang et al.^10^ was ∼3  mm. With our 2.8-mm wide illumination and a spacing between data points of 2 to 3 mm, it should be possible to observe this transmission peak. Utilizing the illumination positions shown in Fig. 2, we analyzed the transmitted signal on two subjects with low and high pigmentation (levels 1 and 5 on the Fitzpatrick scale), shown in Fig. 13. We can observe that the signal level fluctuates by ∼20%; however, we do not observe a highly transparent pars plana, as was reported by Wang et al.^10^ and Toslak et al.^11^. From our experience, the preferred illumination location in our setup is toward the corner of the eye, where signal is high for the two test subjects shown in Fig. 13, and the relatively large separation between illumination and imaging helps reducing the amount of stray light into the imaging system.

### 3.3 Skin Pigmentation and Image Quality

One of the main aims of our study is to investigate and quantify the influence of eye pigmentation on the signal level and the quality of the retina images obtained using the transscleral illumination approach. This will directly affect the applicability of this approach in practice. Previous studies^10^^,^^11^ have not included any detailed discussion on eye pigmentation.

To analyze the influence of pigmentation, a wide range of subjects with different skin pigmentations were recruited. We used the Fitzpatrick scale^18^ to estimate the pigmentation. We used both images taken without a filter and those with a 600-nm shortpass filter. The computed signal levels were normalized with respect to the analog gain.

**Fig. 14:**
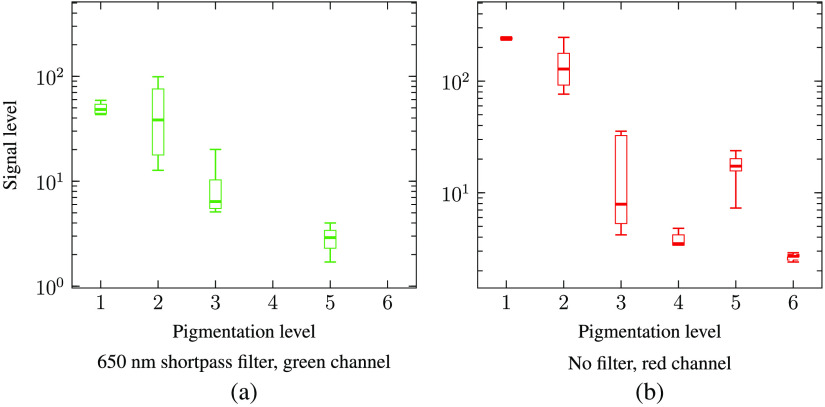
Impact of skin pigmentation on signal level. (a) Signal in green channel, using a 650 nm shortpass filter. (b) Signal in the red channel, without filter. Each range bar shows the minimum, the maximum, the sample median, and the first and third quartiles. The signal level between eyes with low and high pigmentation differed by a factor of 100.

**Fig. 15:**
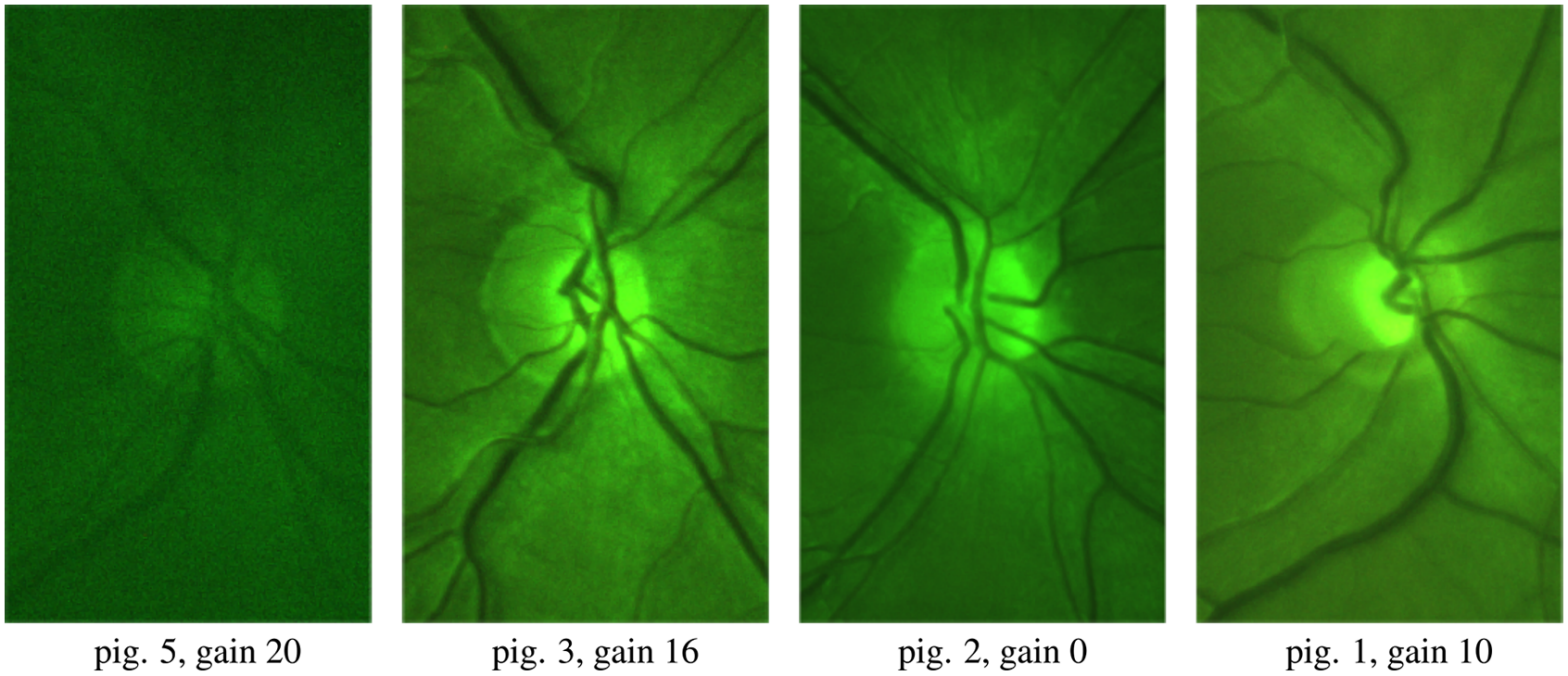
Retina images from subjects with different pigmentation levels. The images are normalized for presentation. Only the most pigmented eyes suffered from insufficient image quality.

**Fig. 16:**
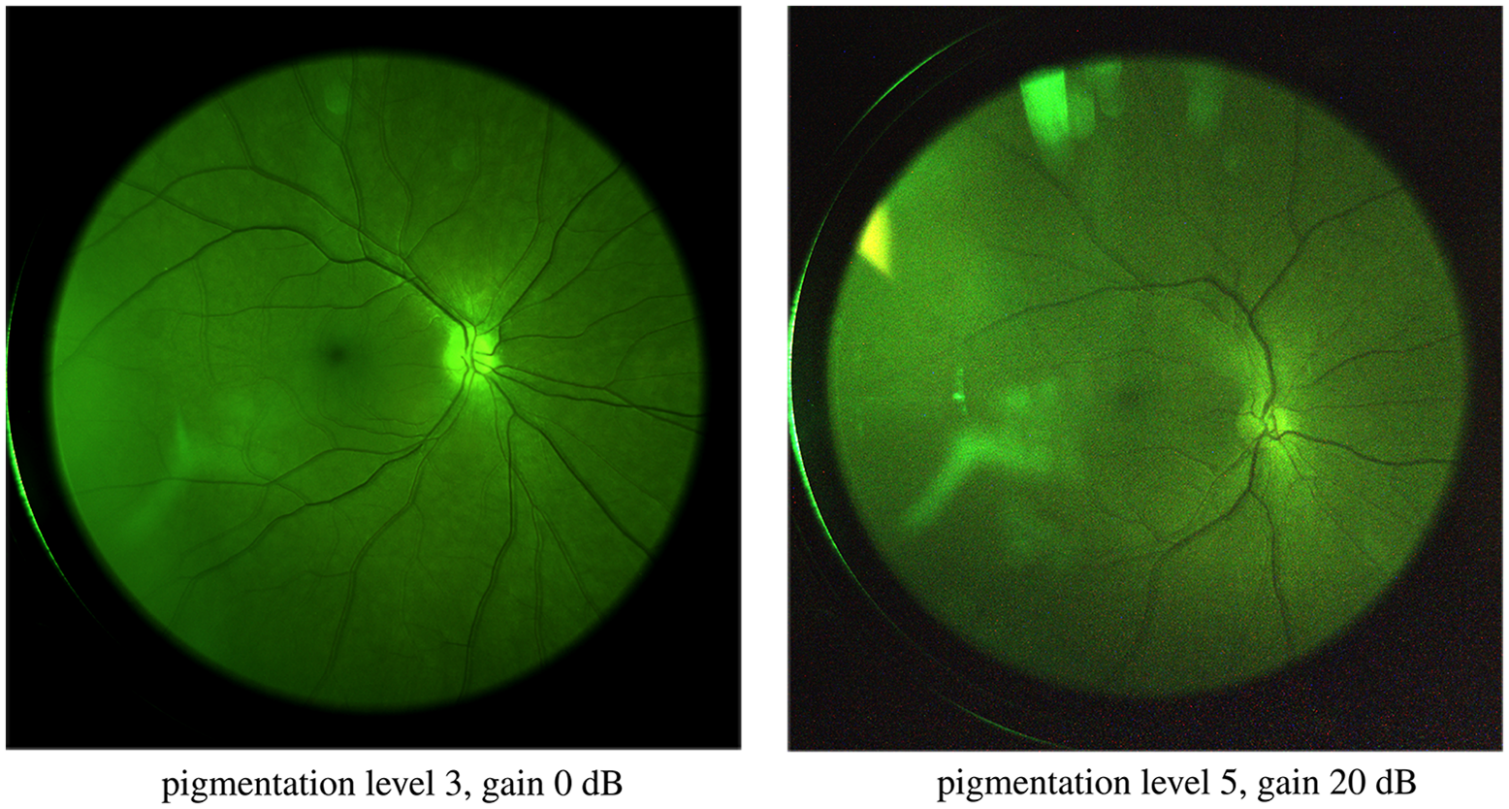
Full-field images from subjects with pigmentation levels 3 and 5. Both images were taken with a 600-nm shortpass filter. Some reflection artefacts are observed in the images.

The results (Fig. 14) show a two orders-of-magnitude difference in the signal levels between subjects of the lowest and highest skin pigmentation. One explanation for the variation in the signal level for subject with different pigmentation levels could be that skin pigmentation (which we assess) does not always correlate well with eye pigmentation. Several actual retina images captured from subjects with different pigmentation are shown in Figs. 15 and 16.

### 3.4 Potential Fundus Camera Design

A versatile fundus camera must be functional regardless of eye pigmentation levels. With our observation of a difference in signal levels depending on eye pigmentation by a factor of 100, it is obvious that a functional transsclera illuminated fundus camera will require some level of adaptive illumination and exposure. A red or infrared pre-flash can be used for both exposure estimation and camera auto-focus. This approach uses lower illumination power, thereby keeping the retinal illumination hazard as low as possible.

Our current design and illumination source are suitable for subjects with low-to-medium pigmentation, but the image quality for highly pigmented eyes is poor (Fig. 15, left) due to low signal levels and a poor signal-to-noise ratio.

A quantitative assessment indicates that signal levels should be above 400 to 800 photoelectrons per pixel (10 to 20 counts) with an illumination range of 500 to 600 nm. This means that the signal must be increased by a factor of 10 to obtain sufficient image quality for highly pigmented eyes. While the exposure time cannot be much higher than the current value of 150 ms (to eliminate the influence of eye movements), it can be seen from Sec. 2.2 that the illumination levels can safely be increased by a factor of 10. Illumination can be increased either by increasing the intensity (higher hazard exposure compared to the current design), or by increasing the illuminated area while keeping intensity constant (same hazard exposure as in the current design).

## 4 Conclusions

A retina camera that utilizes transscleral illumination was constructed and tested on a number of subjects with different pigmentation levels (ranging from 1 to 6 on the Fitzpatrick scale) and using different illumination spectra. Our results provide new insight into the factors impacting practical applicability and design of transcleral fundus cameras.

A high contrast between the blood vessels and retinal background was observed in the images when illuminating with wavelengths in the range of 500 to 600 nm. A detailed analysis of the data demonstrated that the light transmission through the sclera can be approximated by $\alpha e^{\lambda /50}$, where λ is the wavelength of the light source (in nm) and α is a scaling constant.

A difference in the transmitted signal by a factor of 100 was observed between individuals with low and high skin pigmentation. To ensure that the proposed transscleral fundus camera is suitable for both low and high eye pigmentation, the illumination and/or exposure must be adapted to the eyes of each patient.

We have shown that it is possible and eye-safe to use transscleral illumination to obtain high-quality fundus images, even for highly pigmented eyes. In our setup, it is estimated that a tenfold increase in the illumination power will be required to achieve sufficient quality images for highly pigmented eyes. Finally, it was concluded that the illumination wavelengths ranges should range from 500 to 600 nm or 550 to 600 nm to obtain high contrast images, wherein the latter range provides the lowest photochemical impact. Shortpass filtering is particularly relevant, as the high scleral transmission at long wavelengths causes a disproportionate contribution from longer wavelengths of the LED spectrum.

A possible further step is to build a transscleral camera with more powerful illumination and test it on a larger number of subjects with high skin pigmentation, to verify that our suggested improvements provide sufficient image quality.

## Code, Data, and Materials Availability

Data to support the findings can be issued on reasonable request to the corresponding author.
